# Supplementary material for: An open-source framework for large-scale, flexible evaluation of biomedical text mining systems
Source: J Biomed Discov Collab. 2008 Jan 29;3:1. doi: 10.1186/1747-5333-3-1 (PMC2276192; doi:10.1186/1747-5333-3-1)
Supplement: Additional file 1 — Evaluation platform source code. The additional file contains the software evaluation framework discussed in this paper. The most current release can be downloaded from SourceForge.net [1]. See the accompanying README file for instructions on installation and use. Also included in the distribution is a collection of UIMA wrappers for some commonly used BioNLP tools and annotated corpora. [file 1747-5333-3-1-S1.zip › bionlp_uima_repository-1.0.1/build/test/reports/unit/overview-summary.html]

Unit Test Results: Summary


# Unit Test Results

|  |  |
| --- | --- |
|  | Designed for use with JUnit and Ant. |

---

## Summary

| Tests | Failures | Errors | Success rate | Time |
| --- | --- | --- | --- | --- |
| 99 | 0 | 0 | 100.00% | 13.184 |

Note: *failures* are anticipated and checked for with assertions while *errors* are unanticipated.

## Packages

| Name | Tests | Errors | Failures | Time(s) |
| --- | --- | --- | --- | --- |
| edu.uchsc.ccp.uima.cc.summary.comparison | 1 | 0 | 0 | 0.080 |
| edu.uchsc.ccp.uima.util | 12 | 0 | 0 | 5.907 |
| edu.uchsc.ccp.util.external.abner | 2 | 0 | 0 | 4.297 |
| edu.uchsc.ccp.util.external.stanfordparser | 1 | 0 | 0 | 0.009 |
| edu.uchsc.ccp.util.nlp | 9 | 0 | 0 | 0.045 |
| edu.uchsc.ccp.util.nlp.annotation | 26 | 0 | 0 | 0.080 |
| edu.uchsc.ccp.util.nlp.annotation.comparison | 18 | 0 | 0 | 2.355 |
| edu.uchsc.ccp.util.nlp.ei.xml | 2 | 0 | 0 | 0.027 |
| edu.uchsc.ccp.util.nlp.mention | 20 | 0 | 0 | 0.105 |
| edu.uchsc.ccp.util.nlp.mention.comparison | 3 | 0 | 0 | 0.269 |
| edu.uchsc.ccp.util.nlp.stats | 5 | 0 | 0 | 0.010 |
